# Diagnosis of genetic ataxias in adulthood: protocol for a systematic review

**DOI:** 10.1136/bmjopen-2026-124865

**Published:** 2026-10-20

**Authors:** Beatriz Carmona-Hidalgo, Rocío Rodríguez-López, Carola Reinhard, Sylvia Boesch, Anna Sobanska, Elisabetta Indelicato

**Affiliations:** 1 Health Technology Assessment Area-AETSA, Andalusian Public Foundation for Progress and Health, Seville, Spain; 2 Institute for Medical Genetics and Applied Genomics, University of Tübingen, Tubingen, Germany; 3 Centre for Rare Diseases, University Hospital Tübingen, Tübingen, Germany; 4 Center for Rare Movement Disorders, Department of Neurology, Medizinische Universität Innsbruck, Innsbruck, Austria; 5 Institute of Psychiatry and Neurology, Warsaw, Poland

**Keywords:** Systematic Review, Neurogenetics, Protocols & guidelines

## Abstract

**Abstract:**

**Introduction:**

Genetic ataxias are rare neurological diseases with an extremely heterogeneous molecular background. The involvement of more than 100 genes as well as different mutational mechanisms, with a particular abundance of short tandem repeat expansions, represents an intrinsic challenge in the diagnostic process. Moreover, very frequent molecular aetiologies of ataxias with adult onset have only recently been solved. This protocol presents the methodology of a planned systematic review aimed at establishing a standardised diagnostic workflow for genetic ataxias in adulthood, with the aim of optimising their clinical management.

**Methods and analysis:**

The research question in Population, Intervention, Comparison and Outcome format will cover adult patients evaluated via molecular diagnostic techniques. Primary outcomes will focus on the frequency and distribution of molecular diagnoses. Secondary outcomes will evaluate the overall efficiency of the diagnostic process. The search will be performed from 2012 onwards, including systematic reviews, clinical practice guidelines and primary studies. The initial search strategy in MEDLINE (Ovid) is presented. A description of the prospective assessment of the risk of bias in the selected studies and outcomes is provided. A meta-analysis and subgroup analysis will be conducted if the included studies demonstrate sufficient homogeneity and a robust patient sample size. This protocol follows the Preferred Reporting Items for Systematic review and Meta-Analysis Protocols (PRISMA-P) guidelines.

**Ethics and dissemination:**

As this study involves a systematic review of the literature, ethics committee approval is not required. The findings will be disseminated through a peer-reviewed and open-access journal to ensure broad scientific accessibility.

**PROSPERO registration number:**

CRD420261362380.

**STRENGTHS AND LIMITATIONS OF THIS STUDY**

- The protocol adheres to the Preferred Reporting Items for Systematic Reviews and Meta-Analyses for Protocols (PRISMA-P) guidelines.
- A clinical question into Population, Intervention, Comparison and Outcome format was defined to guide the review process: What is the most effective molecular testing algorithm for adults with suspected genetic ataxia to maximise diagnostic yield and the identification of actionable diagnoses?
- Different genetic strategies, frequency and distribution of molecular diagnosis and the overall efficiency of diagnostic procedures will be considered as potential primary and secondary outcomes to evaluate outcomes.
- The methodology includes an assessment of risk of bias across selected studies.
- Additionally, meta-analyses and subgroup analyses will be conducted to identify optimal diagnostic strategies.

## Introduction

 Genetic ataxias are a heterogeneous group of rare neurological diseases caused by damage to the cerebellum and/or its connections.^1^ Genetic ataxias share a common clinical presentation with balance and coordination disturbances which have typically an insidious onset and, in the majority of cases, a chronic progressive course.^1^ Even if each single disease entity is rare, the pooled prevalence of genetic ataxias in Europe was estimated to reach 11.2 cases per 10 000 persons in 2014.^2^ The true estimate is likely to be much higher. In fact, recent advances in molecular genetics have shown that several sporadic ataxias previously classified as ‘idiopathic’ do have a genetic cause.^3^ Raising awareness and accelerating the time to diagnosis are priority objectives in health policy and research focused on people living with rare diseases.^4 5^ Diagnostic delay and limited awareness can lead to a series of referrals, prescription of redundant tests, prolonged diagnostic processes, limited treatment options and ultimately lack of coordinated care and increased health and social costs/burden.^6^ In genetic ataxias, the choice of optimal diagnostic procedures is complicated by the pleiotropy of more than 100 ataxia genes, the wide phenotypic variability and the multiple underlying mutational mechanisms, with a particularly high number of short tandem repeat expansions, which require prioritisation of genetic testing strategies. In addition, two of the most common molecular aetiologies of ataxia with adult onset (*RFC1* and GAA-*FGF14* disease) have only recently been described.^7 8^ Genetic diagnostic practices vary widely across Europe due to financial and availability issues. Nevertheless, first-line diagnostics in genetic ataxias can be performed with widely available, low-cost diagnostic tools before applying next-generation sequencing or long-read sequencing. In the clinical fields, there is a lack of awareness of this concept.^9 10^ All these issues are sources of uncertainty about best practice in this area. Notably, the last official European consensus paper was published more than 10 years ago.^11^ In terms of clinical management, there has been some exciting news in recent years. The first treatment for one of the most common genetic ataxias, Friedreich’s ataxia, was approved in the European Union in 2024, making early diagnosis imperative.^12^ Evidence of efficacy has also accumulated in recent years for several other therapeutic approaches (eg, 4-aminopyridine in GAA-*FGF14* ataxia, N-acetyl-l-leucine in Niemann-Pick type C disease),^13 14^ demanding a prompt diagnosis as well. Considering all these issues, the development of an updated diagnostic algorithm for genetic ataxias in adulthood has been endorsed by the European Reference Network for Rare Neurological Diseases (ERN-RND). Furthermore, this initiative has been granted a priority designation in the guidelines programme by the European Academy of Neurology. Herein, we present the protocol for the systematic review of the literature which will support this document.

## Methods and analysis

This protocol follows the Preferred Reporting Items for Systematic review and Meta-Analysis (PRISMA) Protocols guidelines^15^ specified in online supplemental data 1. The final systematic review will be conducted according to the PRISMA statement.^16^

### Eligibility criteria

The goal of this systematic review is to answer the following research question in PICO format (Population Intervention Comparator Outcome): What is the most effective molecular testing algorithm for adults with suspected genetic ataxia to maximise diagnostic yield and the identification of actionable diagnoses? (table 1). To this end, we will review the available literature for studies addressing the molecular diagnosis of adult patients whose clinical presentation is consistent with genetic ataxia, as described in detail under the ‘Population’ criterion in table 1. This means that either other aetiologies of ataxia have been previously excluded or the clinical presentation supports a genetic origin of the disease. Included are presentations with chronic or progressive cerebellar or spinocerebellar syndromes, spastic ataxia and episodic cerebellar syndromes. We will include patients irrespective of the age at onset of symptoms. Paediatric patients will be excluded. Considering the ‘Intervention’ criterion, to ensure comprehensive coverage of diagnostic assessments, we will consider various genetic testing strategies including repeat expansion detection techniques, next-generation sequencing-based techniques (panels, whole exome sequencing, whole genome sequencing), copy number variant analysis, multiplex ligation-dependent probe amplification assay and long-read sequencing. Comparators will not be considered. During our search, we will analyse the frequency and distribution of molecular diagnoses as the primary outcome and we will collect specific clinical characteristics, to evaluate whether they are associated with specific molecular diagnoses. Indeed, given the current lack of consensus in diagnostic criteria*,* our aim is to help clinicians identify the most common genetic aetiologies to consider based on specific clinical presentations, thus defining a diagnostic algorithm. We do not aim to evaluate the performance of one diagnostic technique versus others because the choice of analytical technique in daily practice is influenced by factors independent of clinical suspicion, such as financial considerations and availability. Furthermore, to the best of our knowledge, comparisons of the diagnostic yield of different techniques are scarce. However, if feasible based on the collected literature, we will evaluate the efficiency of the diagnostic procedures through related parameters such as the time to molecular diagnosis and estimated costs as secondary outcomes.

**Table 1:** Population, intervention, comparison and outcomes criteria of the PICO question defined for the planned systematic review

PICO question	What is the most effective molecular testing algorithm for adults with suspected genetic ataxia to maximise diagnostic yield and the identification of actionable diagnoses?
Population	Adults (≥18 years old at evaluation) with clinical presentation compatible with a genetic cause of ataxia*, including: Chronic/progressive cerebellar syndrome. Chronic/progressive spinocerebellar syndrome. Chronic/progressive spastic ataxia. Episodic cerebellar syndrome. *After exclusion of secondary causes whenever appropriate
Intervention	Genetic testing. Included, but not limited to: Repeat expansion detection techniques. Next-generation sequencing based techniques (panels, whole exome sequencing, whole genome sequencing). Copy number variant analysis. Multiplex ligation-dependent probe amplification. Long-read sequencing.
Comparator	None
Outcomes	Primary outcome: Molecular diagnosis. Overall rate of confirmed genetic diagnosis and specific molecular findings: Gene. Variant. Frequency of specific molecular diagnoses. Type and frequency of actionable molecular diagnoses. Secondary outcomes: Efficiency of the diagnostic process, including: Time to molecular diagnosis Estimated costs Organisational features/personnel.

Considering studies characteristics and PICO framework previously described, articles will be eligible for inclusion if they are systematic reviews, clinical practice guidelines (CPGs) and primary studies (case series), excluding narrative reviews, conference abstracts, editorials and letters to the editor. We will exclude case reports and case series describing less than 10 patients or one family only. Furthermore, we will exclude studies addressing mixed population (eg, various movement disorders/neurogenetic disorders including also ataxias). Studies with incomplete data and those whose full text is unavailable will also be excluded. This protocol has been previously registered in the International Prospective Register of Systematic Reviews (PROSPERO) repository with the identification CRD420261362380.

### Search strategy

The searches will be carried out in the following core and subject-specific databases: MEDLINE (Ovid), Embase (Elsevier), the Cochrane Database, TRIP Database, ECRI Guidelines, Gene Reviews, NORD (National Organisation of Rare Disorders), ORPHANET, EURORDIS and Espistemonikos. We will consider as further bibliographic resources: the Web of Science Core Collection (WOS), Uptodate and INAHTA database. The searches will include both controlled language (descriptors) and free text.^17^ The search terms are related to ataxia as disease and the identified genes and the variants. Regarding the intervention, the terms are related to genetic techniques. The literature search will be restricted to English-language publications from 2012 onwards. This timeframe was selected because the most recent CPG regarding the diagnosis and treatment of adult genetic ataxias covers evidence up to 2012.^11^ The preliminary search, developed in MEDLINE (Ovid), is available in online supplemental data 2. This search will be adapted to the rest of databases.

### Article selection

The screening process will be carried out through different phases following the PRISMA

guidelines.^16^ The retrieved records from the literature search will be imported to Covidence, a systematic review data management platform (https://www.covidence.org) and the duplicates will be removed. Two reviewers will independently screen the titles and abstracts, and after that, perform the full-text screening. Any disagreements will be resolved by discussion to achieve a consensus. If it is not achieved, a third reviewer will be consulted (figure 1).

**Figure 1:**
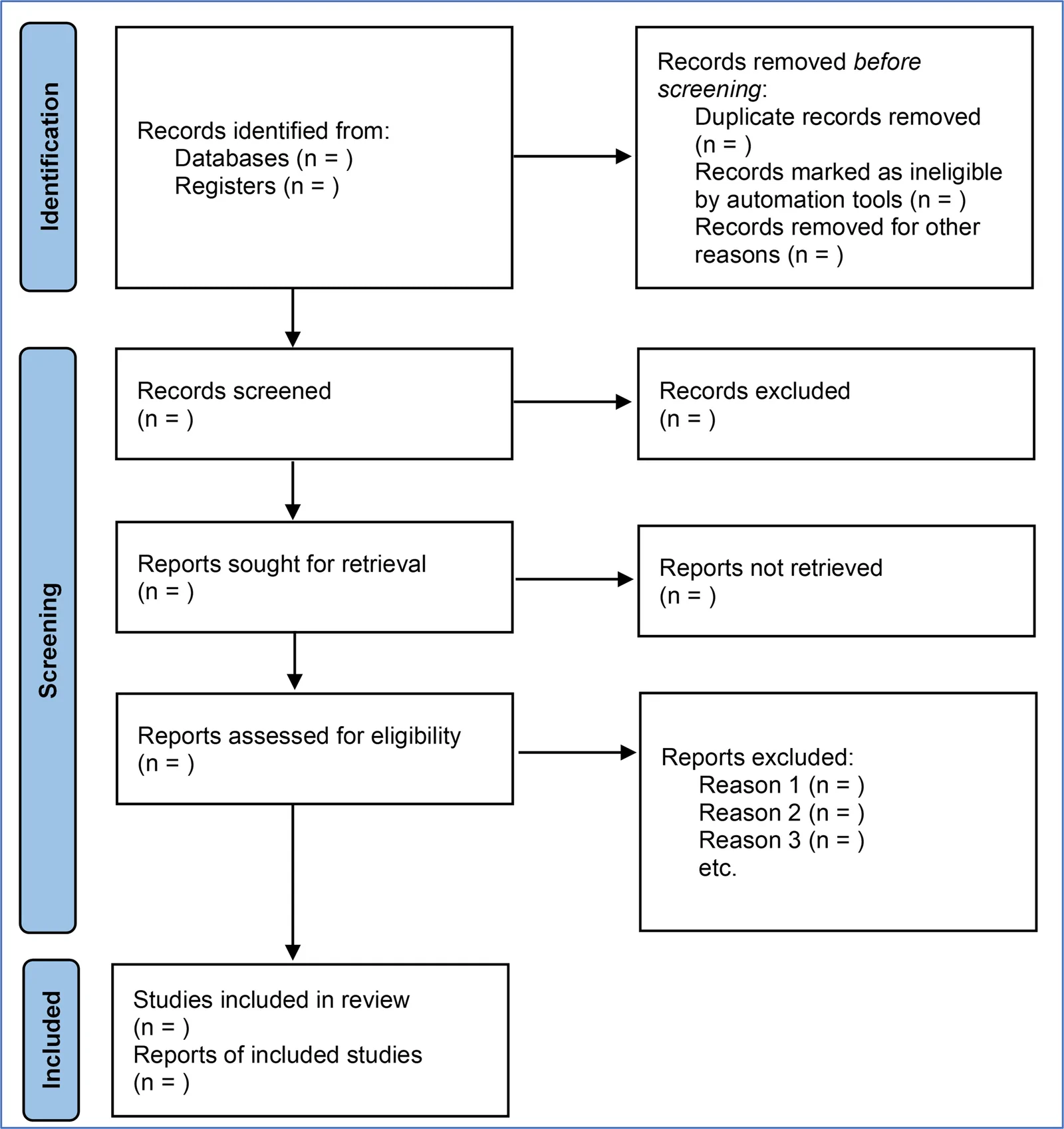
shows the flow diagram of the study selection process.

### Data extraction

The full text included articles will be assessed by two independent reviewers to extract the data. Any discrepancies between reviewers will be discussed and resolved by an additional reviewer. The data extraction will be done in Covidence. The data to be extracted will include specific details of the studies (author, year of publication, study type, location), the patients (age, gender, key clinical features, previous genetic screening), intervention (type of genetic tests) and outcomes (genetic variants, inheritance, actionable molecular diagnosis, among others). It is expected to extract individual patient data. If necessary, study authors will be contacted to request any necessary missing information. A priori extraction template is presented in table 2.

**Table 2:** Preliminary overview of the data to be extracted from the selected studies for the planned systematic review

Article data	Article type
	Title
	Year
	Authors
	Objective
	Country
Patient data	Number of patients
	Gender distribution
	Age at examination
	Age at ataxia onset
	Clinical presentation
	Inheritance
	Neuroimaging findings (cerebellar atrophy at brain MRI)
	Electrophysiological findings (presence of neuropathy)
	Findings from other examinations
Intervention	Genetic techniques
Outcomes	Frequency of molecular diagnoses
	Type of diagnoses (gene)
	Detailed molecular findings (variant)
	Diagnostic flowchart
	Organisational aspects
	Treatment strategy
	Clinical usefulness

### Outcomes

We will evaluate the overall rate of confirmed genetic diagnosis and the frequency of specific molecular diagnoses, with a focus on the actionable ones. Efficiency of the diagnosis procedure will be analysed through related parameters as time, costs and organisational features. Quality of life indicators, safety and adverse events related to the diagnosis may also emerge during the literature review and data analysis.

### Risk of bias and quality of articles

The quality of every selected reference will be assessed using different evaluation tools depending on the type of article. For systematic reviews, risk of bias will be assessed using the AMSTAR-II tool^18^; AGREE-II tool will be used for CPG,^19^ and the tool developed by Murad *et al*^20^ for case series. The assessment will be performed by two authors independently and the disagreements will be resolved by discussion. If an agreement is not achieved, a third reviewer will participate. The risk of bias assessment results will be summarised in tables and text in the completed review. The quality of the outcomes will be assessed and the results summarised by applying the methodology developed by the Grading of Recommendations, Assessment, Development and Evaluation System (GRADE) tool to adopt or adapt the recommendations.^21^

### Data synthesis

Information about patients and study characteristics will be collected and presented in a tabular form. If there will be enough studies to quantitatively synthesise the results, heterogeneity between studies will be calculated using the I^²^ statistic. Random effects meta-analysis will be conducted with 95% CIs. If possible, pooled estimates of OR/RR (risk ratios) with 95% CIs will be calculated. If the sample of patients is sufficiently large, this information could be separated in subgroups, for example, as defined by the age at onset of ataxia (early ≤25 years, vs late ≥40), presence of cerebellar atrophy at MRI, presence of episodic features, type of inheritance (autosomal dominant, autosomal recessive, negative family history), neuropathy (presence and type) or other relevant factors and outcomes. The analyses will be conducted with RevMan (https://training.cochrane.org/online-learning/core-software/revman), a systematic review and meta-analysis tool designed by Cochrane. If studies are too heterogeneous or there is a lack of reported data,^22^ a narrative synthesis of the findings will be performed.

### Patient and public involvement

Patient representatives are involved in the design, conduct, reporting and dissemination of this research.

### Study timeline

The systematic review project was initiated in September 2026. We plan to complete the article selection, data extraction and quality assessment within the first quarter of 2027 and the data synthesis and analysis within the second quarter. Subsequently, a set of recommendations for a diagnostic algorithm will be formulated by the expert clinicians of the ERN-RND.

## Discussion

This review aims to provide systematic evidence as basis for an update of the current diagnostic algorithm for genetic ataxias in adulthood. Several reviews in the past years have addressed-related aspects such as the epidemiology of genetic ataxias,^2 23^ the yield of genetic examinations in respect to different testing strategies,^24^ the modality of genetic testing and counselling.^25^ However, most of this literature did not specifically address the need of an updated, comprehensive, evidence-based diagnostic algorithm concerning genetic ataxia in adulthood. In this regard, the last official European guideline on the topic dates back to 2014.^11^ A more recent guideline has been released by the patient organisation Ataxia UK in 2019 and received endorsement also by ERN-RND.^26^ This resource, which has been created by a UK only panel list, does not cover genetic forms with episodic course. The recommendation for genetic testing is based on logistical considerations related to the referral and reimbursement practices in the UK. Furthermore, several milestones in diagnosis and therapy have been set in the five last years. Therefore, an updated guideline in the field is urgently needed. This review explicitly covers ataxias in adulthood and not ‘ataxias with an adult onset’ for several reasons relevant as to the clinical practice, as previously pursued.^11^ Many patients first present themselves at an adult age to the neurologists but retrospectively describe an onset of ataxia before the age of 18. In these cases, the differential diagnosis needs to contemplate disorders that manifest at these earlier ages. Furthermore, several diseases have a broad age at onset range.^27^ Of note, clinical recommendations for the diagnosis of ataxias in children have been already covered by a previous consensus publication endorsed by ERN-RND.^28^ A limitation, which may emerge, could be represented by insufficient evidence in the literature. Heterogeneity can be anticipated, in terms of type of studies, background of the patients and the quantity and quality of data reported. There could also be limitations in the specification of type of genetic tests and in the workflow established in their use as diagnostic procedure. We anticipate that information concerning age at onset and the clinical phenotype may be incomplete, an issue that may affect the planned subanalyses. Considering that the diagnostic testing algorithms applied in the literature are heterogeneous, this may introduce a bias concerning our assumption about the frequency of specific molecular diagnoses. In addition, it could be challenging to compare the results across both quantitative and qualitative data. To address these limitations, we might analyse subgroups of patients with similar characteristics. Articles could also be grouped by type of study (systematic reviews, clinical practice guidelines, primary studies). Finally, our focus is on genetic ataxias caused by gene variants reported in the existing literature, but the landscape of known genes associated with ataxia is continuously evolving. For this reason, our workflow is optimised to allow for ongoing updates. Given the ERN-RND’s commitment to improving the care of patients with RNDs, the present project fully fits with this goal. We anticipate that the findings from this review will refine the standards of care, accelerate diagnosis and potentially improve access to therapy for adult patients with ataxia.

## Ethics and dissemination

Considering the nature of the study, ethics committee approval is not required. For the same reason consent to participate does not apply. The results will be disseminated through a peer-reviewed open access publication due to the potential to impact on the clinical management of this condition. Any deviations from this protocol will be recorded and explained in the final study.

## Supplementary material

10.1136/bmjopen-2026-124865 online supplemental file 1

10.1136/bmjopen-2026-124865 online supplemental file 2
