# Supplementary material for: Diagnosis of genetic ataxias in adulthood: protocol for a systematic review
Source: BMJ Open. 2026 Oct 20;16(10):e124865. doi: 10.1136/bmjopen-2026-124865 (PMC13684238; doi:10.1136/bmjopen-2026-124865)
Supplement: Supplementary file 1 [file bmjopen-2026-124865.reviewer_comments.pdf]

PEER REVIEW HISTORY

BMJ Open publishes all reviews undertaken for accepted manuscripts. Reviewers are asked to complete a checklist review form and are provided with free text boxes to elaborate on their assessment. These free text comments are reproduced below.

ARTICLE DETAILS

Title (Provisional)

DIAGNOSIS OF GENETIC ATAXIAS IN ADULTHOOD: PROTOCOL FOR A SYSTEMATIC REVIEW

Authors

Carmona-Hidalgo, Beatriz; Rodríguez-López, Rocío; Reinhard, Carola; Boesch, Sylvia; Sobanska, Anna; Indelicato, Elisabetta; ERN-RND, European Reference Network for RNDs

---

VERSION 1 - REVIEW

**Reviewer**	**1**
**Name**	**Inagawa, Takuma**
**Affiliation**	**National Center of Neurology and Psychiatry, National Center of Neurology**
**Date**	**20-Aug-2026**
**COI**	**None**

Thank you for giving me the opportunity to review this manuscript.

1) Please describe the inclusion and the exclusion criteria more in detail to understand that these criteria fully answer the question of "What is the most effective

molecular testing algorithm for adults with suspected genetic ataxia to maximize

diagnostic yield and the identification of actionable diagnoses?".

2) Please describe the study design of each study to extract.

3) The authors described that we will evaluate the overall rate of confirmed genetic diagnosis, the frequency of specific molecular diagnoses, with a focus on the actionable ones and the most frequent genetic strategies used. Please describe what kind of diagnostic criteria will be selected.

I think it is better to revise the manuscript.

VERSION 1 - AUTHOR RESPONSE

Reviewer: 1
Dr. Takuma Inagawa, National Center of Neurology and Psychiatry
Comments to the Author:
Thank you for giving me the opportunity to review this manuscript.

Comment 1) Please describe the inclusion and the exclusion criteria more in detail to understand that these criteria fully answer the question of "What is the most effective molecular testing algorithm for adults with suspected genetic ataxia to maximize diagnostic yield and the identification of actionable diagnoses?".

Response:

We thank the reviewer for this important remark. In the paragraph "Eligibility Criteria", we now describe the inclusion and exclusion criteria that we will apply to answer the PICO question in more detail (see the added text marked in yellow below).

ELIGIBILITY CRITERIA

The goal of this systematic review is to answer the following research question in PICO format (Population-Intervention-Comparator-Outcome): What is the most effective molecular testing algorithm for adults with suspected genetic ataxia to maximize diagnostic yield and the identification of actionable diagnoses? (Table 1). To this end, we will review the available literature for studies addressing the molecular diagnosis of adult patients whose clinical presentation is consistent with genetic ataxia, as described in detail under the "Population" criterion in Table 1. This means that either other etiologies of ataxia have been previously excluded or the clinical presentation supports a genetic origin of the disease. Included are presentations with chronic or progressive cerebellar or spinocerebellar syndromes, spastic ataxia, and episodic cerebellar syndromes. We will include patients irrespectively of the age at onset of symptoms. Pediatric patients will be excluded. Considering the "Intervention" criterion, to ensure a comprehensive coverage of diagnostic assessments, we will consider various genetic testing strategies including repeat expansion detection techniques, next-generation sequencing based techniques (panels, whole exome sequencing, whole genome

sequencing), copy number variant analysis, multiplex ligation-dependent probe amplification assay and long-read sequencing. Comparators will not be considered. During our search, we will analyse the frequency and distribution of molecular diagnoses as the primary outcome and we will collect specific clinical characteristics, to evaluate whether they are associated with specific molecular diagnoses. Indeed, our aim is to help clinicians identify the most common genetic etiologies to consider based on specific clinical presentations, thus defining a diagnostic algorithm. We do not aim to evaluate the performance of one diagnostic technique versus others because the choice of analytical technique in daily practice is influenced by factors independent of clinical suspicion, such as financial considerations and availability. Furthermore, to the best of our knowledge, comparisons of the diagnostic yield of different techniques are scarce. However, if feasible based on the collected literature, we will evaluate the efficiency of the diagnostic procedures through related parameters such as the time to molecular diagnosis and estimated costs as secondary outcomes.

Considering studies characteristics, articles will be eligible for inclusion if they are systematic reviews, clinical practice guidelines (CPGs) and primary studies (case series), excluding narrative reviews, conference abstracts, editorials and letters to the editor. We will exclude case reports and case series describing less than 10 patients or one family only. Furthermore, we will exclude studies addressing mixed population (e.g. various movement disorders/neurogenetic disorders including also ataxias). Studies with incomplete data and those whose full text is unavailable will also be excluded. This protocol has been previously registered in the International Prospective Register of Systematic Reviews (PROSPERO) repository with the identification CRD420261362380.

Comment 2) Please describe the study design of each study to extract.

Response:

Thank you for your comment. As described in the methodology, based on the PICO framework, the evidence to be extracted will be derived from systematic reviews, clinical practice guidelines, and primary studies with more than 10 patients to minimize selection bias regarding the population and intervention.

To this regard, we addressed it more in details in the paragraph eligibility criteria:

Considering studies characteristics and PICO framework previously described, articles will be eligible for inclusion if they are systematic reviews, clinical practice guidelines (CPGs) and primary studies (case series), excluding narrative reviews, conference abstracts, editorials and letters to the editor.

Comment 3) The authors described that we will evaluate the overall rate of confirmed genetic diagnosis, the frequency of specific molecular diagnoses, with a focus on the actionable ones and the most frequent genetic strategies used. Please describe what kind of diagnostic criteria will be selected.

Response:

We thank the reviewer for this important remark. First of all, we corrected the sentence cited by the reviewer, which, a posteriori, was not clear:

We will evaluate the overall rate of confirmed genetic diagnosis and the frequency of specific molecular diagnoses, with a focus on the actionable ones ~~and the most frequent genetic strategies used.~~

Currently, there are no up-to-date, internationally aligned diagnostic criteria for genetic ataxias. The type of genetic testing chosen is based on clinical considerations, which continuously change based on knowledge updates, as well as on logistical criteria based on the availability of certain types of genetic testing in individual centers.

Thus, based on this review, our aim is to define a diagnostic algorithm that suggests to the clinician the most likely molecular etiologies to be considered based on the clinical setting. We have clarified this important point in the text:

During our search, we will analyse the frequency and distribution of molecular diagnoses as the primary outcome and we will collect specific clinical characteristics, to evaluate whether they are associated with specific molecular diagnoses. Indeed, given the current lack of consensus in diagnostic criteria, our aim is to help clinicians identify the most common genetic etiologies to consider based on specific clinical presentations, thus defining a diagnostic algorithm. We do not aim to evaluate the performance of one diagnostic technique versus others because the choice of analytical technique in daily practice is influenced by factors independent of clinical suspicion, such as financial considerations and availability.

VERSION 2 - REVIEW

Reviewer 1

Name Inagawa, Takuma

Affiliation National Center of Neurology and Psychiatry, National Center of Neurology

Date 24-Sep-2026

COI

I think this manuscript would be suitable for publication.
