# Supplementary material for: Diagnosis of genetic ataxias in adulthood: protocol for a systematic review
Source: BMJ Open. 2026 Oct 20;16(10):e124865. doi: 10.1136/bmjopen-2026-124865 (PMC13684238; doi:10.1136/bmjopen-2026-124865)
Supplement: Supplementary file 3 [file bmjopen-16-10-s002.docx]

**Supplementary Data 2.** Preliminary search strategy in MEDLINE database.

1 Ataxia/

2 exp Spinocerebellar Degenerations/

3 ((Cerebell* or spinocerebell* or mitochondrial* or spastic* or Friedreic* or genetic or inherit* or famili* or hereditar* or congenital* or autosomal or dominant or recessive or x linked or x associated or SCA* or ATX* or FGF* or FMR1* or CACNA* or TBP* or PPPR2R2B* or NOP56* or BEAN1* or DAB1* or ATN1*) and (Ataxia* or ataxy*)).ti,kf.

4 ((Cerebell* or spinocerebell* or mitochondrial* or spastic* or Friedreic* or genetic or inherit* or famili* or hereditar* or congenital* or autosomal or dominant or recessive or x linked or x associated or SCA* or ATX* or FGF* or FMR1* or CACNA* or TBP* or PPPR2R2B* or NOP56* or BEAN1* or DAB1* or ATN1*) adj3 (Ataxia* or ataxy*)).ab.

5 (machado* adj3 (atax* or disease*)).ti,ab,kf.

6 1 or 2 or 3 or 4 or 5

7 exp Genetic Techniques/

8 ((gene* or multigene* or chromosoma* or delet* or duplicat* or "copy number" or CNV* or DNA* or RNA* or mRNA* or protein* or molecul* or Sanger* or "single nucleotide variants" or SNV* or "second generation" or "next generation" or "third generation" or "long read" or nanopore* or "real time" or exome* or genome* or NKX2* or multiplex* or cytogenet* or expansion* or permutation*) adj4 (test* or screen* or techniq* or analys* or panel* or array* or assay* or microarray* or stud* or sequenc* or amplification*)).ti,kf,ab.

9 (Karyotyp* or "Polymerase Chain Reaction" or PCR* or qPCR* or WES* or WGS* or MLPA* or CMA* or NGS* or TGS* or Southern*).ti,ab,kf.

10 7 or 8 or 9

11 6 and 10

12 (systematic review or meta-analysis).pt.

13 meta-analysis/ or systematic review/ or systematic reviews as topic/ or meta-analysis as topic/ or "meta analysis (topic)"/ or "systematic review (topic)"/ or exp technology assessment, biomedical/ or network meta-analysis/

14 (((systematic* or scoping*) adj3 (review* or overview*)) or (methodologic* adj3 (review* or overview*))).ti,ab,kf.

15 ((quantitative adj3 (review* or overview* or synthes*)) or (research adj3 (integrati* or overview*))).ti,ab,kf.

16 ((integrative adj3 (review* or overview*)) or (collaborative adj3 (review* or overview*)) or (pool* adj3 analy*)).ti,ab,kf.

17 (data synthes* or data extraction* or data abstraction*).ti,ab,kf.

18 (handsearch* or hand search*).ti,ab,kf.

19 (mantel haenszel or peto or der simonian or dersimonian or fixed effect* or latin square*).ti,ab,kf.

20 (met analy* or metanaly* or technology assessment* or HTA or HTAs or technology overview* or technology appraisal*).ti,ab,kf.

21 (meta regression* or metaregression*).ti,ab,kf.

22 (meta-analy* or metaanaly* or systematic review* or biomedical technology assessment* or bio-medical technology assessment*).mp,hw.

23 (medline or pubmed or medlars or cochrane or embase or cinahl or psycinfo or wos or 'web of science' or scopus).ti,ab,hw.

24 (cochrane or (health adj2 technology assessment) or evidence report).jw.

25 (comparative adj3 (efficacy or effectiveness)).ti,ab,kf.

26 (outcomes research or relative effectiveness).ti,ab,kf.

27 ((indirect or indirect treatment or mixed-treatment or bayesian) adj3 comparison*).ti,ab,kf.

28 (multi* adj3 treatment adj3 comparison*).ti,ab,kf.

29 (mixed adj3 treatment adj3 (meta-analy* or metaanaly*)).ti,ab,kf.

30 umbrella review*.ti,ab,kf.

31 (multi* adj2 paramet* adj2 evidence adj2 synthesis).ti,ab,kf.

32 (multiparamet* adj2 evidence adj2 synthesis).ti,ab,kf.

33 (multi-paramet* adj2 evidence adj2 synthesis).ti,ab,kf.

34 or/12-33

35 11 and 34

36 (("historical article" or comment or editorial or news) not "case report*").ti,pt.

37 animals/ not humans/

38 (adolescent/ or exp child/ or exp infant/ or birth cohort/) not exp Adult/

39 35 not (36 or 37 or 38)

40 limit 39 to yr="2012 - 2026"
